# *Colletotrichum fructicola* CfGti1 Transcriptionally Regulates Penetration, Colonization, and Pathogenicity on Apple

**DOI:** 10.3390/jof12010036

**Published:** 2026-01-02

**Authors:** Wenkui Liu, Wei Zhang, Wenxin Shi, Yecan Pan, Pengbo Dai, Chen Yang, Yanjie Wang, Mark L. Gleason, Rong Zhang, Guangyu Sun, Bianqing Hao

**Affiliations:** 1 Shanxi Center for Testing of Functional Agro-Products, Shanxi Agricultural University, Taiyuan 030031, China; 2 State Key Laboratory of Crop Stress Biology in Arid Areas, Northwest A&F University, Yangling, Xianyang 712100, China; 3 College of Plant Protection, Northwest A&F University, Yangling, Xianyang 712100, China; 4 Department of Plant Pathology and Microbiology, Iowa State University, Ames, IA 50011, USA

**Keywords:** glomerella leaf spot, pathogenesis, Gti1, transcription factor, apple

## Abstract

Glomerella leaf spot (GLS), mainly caused by *Colletotrichum fructicola*, is a destructive disease of apple. However, the underlying pathogenesis mechanisms of GLS are still largely obscure. Previous infection transcriptome analysis showed that transcription factor CfGti1 was induced during leaf infection. The present study confirms that the *CfGti1* gene is strongly expressed in conidia and early infection. To identify functions performed, we generated gene deletion mutant Δ*CfGti1* by homologous recombination. Phenotypic analysis revealed that Δ*CfGti1* lost pathogenicity to apple leaves by blocking appressorium-mediated host penetration, although penetration pegs still developed on cellophane. In addition, Δ*CfGti1* colonization and hyphal extension in wounded apple fruit were dramatically decreased. The Δ*CfGti1* mutant exhibited defects in growth and development of hyphae, which may be partly responsible for its inability to colonize apple. Comparative transcriptome and qRT-PCR analyses suggested that CfGti1 regulated appressorium-mediated host penetration by modulating genes related to metabolism of appressorial lipid droplets. Interestingly, CfGti1 also regulated the expression of *ybtS* and *AKT1* or *AFT1-1* related to biosynthesis of AK and AF host-specific toxins. This study demonstrated that CfGti1 is a pivotal regulator for apple GLS pathogenesis in *C. fructicola*.

## 1. Introduction

Glomerella leaf spot (GLS) is a severe foliar and fruit disease harming apple production. In periods of high temperature and humidity, GLS leads to severe leaf and fruit spots, defoliation, and weakening of the trees [1,2]. In the past 30 years, GLS has spread rapidly to Brazil, America, China, Japan, and Uruguay [1,3,4,5]. In China, GLS was first found in 2011 in Feng County, Jiangsu, and caused severe defoliation in 90% of Gala and Golden Delicious cultivars [5]. GLS has spread to most apple-producing areas and has become an epidemic fungal disease in China due to its rapid development and difficult control [2].

Multiple species of *Colletotrichum* have been reported as causal agents of glomerella leaf spot (GLS). These include species from the Gloeosporioides section, such as *C. gloeosporioides*, *C. aenigma*, *C. fructicola*, *C. asianum*, and *C. chrysophilum*, as well as *C. karstii* from the Boninense section [4,6,7,8,9,10,11,12,13]. Of these, *Colletotrichum fructicola* is one of the predominant pathogens worldwide.

*C. fructicola* is a widely distributed fungus causing leaf black spot or fruit rot disease on a broad range of host flora (>90) including cherry, apple, pear, kiwifruit, strawberry, and tea oil [14,15,16,17,18]. As a hemibiotrophic fungus, the *C. fructicola* differentiates into specialized infection structures—appressoria to penetrate host epidermal cell, then bulbous biotrophic hyphae developed inside living epidermal cells, followed by thin, highly destructive necrotrophic hyphae formed to kill and degrade host tissues [19]. Infection-related structure and morphological changes are essential for infection and indicate the changes during the infection stage [19,20,21].

Identification and functional studies of pathogenic factors in GLS pathogens, mostly in *C. fructicola*, have gradually attracted researchers’ attention in recent years. In *C. fructicola*, a histone deacetylase Cfhos2 and a mitogen-activated protein kinase (MAPK) CfPMK1 are required for appressorium formation and pathogenicity [22,23]. MAPK downstream transcription factor CfSte12 facilitates GLS pathogenicity by impacting conidial germination, appressorium formation, and penetration [24]. A recent study found that the MADS-box protein CfMcm1 of the GLS pathogen *C. fructicola* is an indispensable transcription factor for appressorium development during infection [25]. In *C. gloeosporioides*, the ATP-binding cassette protein CgABCF2 and a carbamoyl phosphate synthase subunit CgCPS1 were both involved in appressorial formation, growth, and pathogenicity [26,27]. In addition, an effector Sntf2 of GLS pathogen *C. gloeosporioides* was found to promote invasion by suppressing plant defense responses including callose deposition and H_2_O_2_ accumulation [28]. In *C. fructicola* causing tea-oil anthracnose, several pathogenic factors were identified. Two retromer complex proteins, CfVps35 and CfVps29, of *C. fructicola* participated in functional appressorium formation and pathogenicity [29,30]. A histone acetyltransferase CfGcn5 is essential for appressorium formation and pathogenicity by negatively regulating autophagy [31,32]. Furthermore, three endoplasmic reticulum stress-response-related proteins were identified, which are a SANT-domain-containing protein CfSnt2 that regulates pathogenicity, autophagy, and responses to oxidative stress, and a bZIP transcription factor CfHac1 and a SNARE protein CfVam7 that are both important in growth, appressorium formation, and pathogenicity, respectively [33,34,35].

Our previous comparative transcriptome analysis of leaf infection revealed that the *CfGti1* gene of *C. fructicola* was strongly induced during *C. fructicola* infection [14]. Based on hypothesized GLS pathogenesis mechanisms, CfGti1 was characterized in *C. fructicola* in the present study. The results showed that CfGti1 transcriptionally regulates pathogenicity and colonization by influencing development of penetration pegs and hyphae.

## 2. Materials and Methods

### 2.1. Bioinformatic Analysis

**Figure 1:**
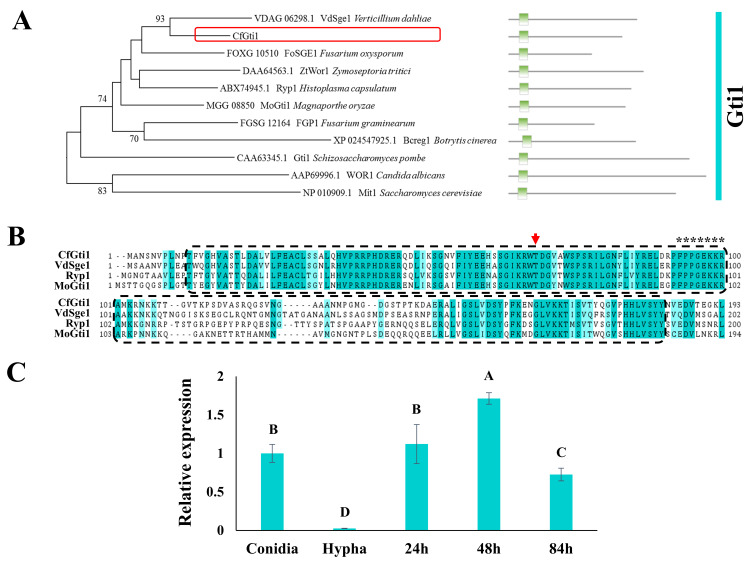
Phylogenetic and structural domain analysis and gene expression pattern analysis. (**A**): Phylogenetic analysis of CfGti1 and Gti1 orthologs in selected fungi. The corresponding sequence length and domain location are shown on the right. Gti1/Pac2 domains are indicated as green rectangles. The phylogenetic tree was constructed using 1000 non-parametric bootstrap runs with the neighbor-joining method; bootstrap percentages over 50% are indicated at the nodes. (**B**): Multiple amino acid sequence alignments of the N-terminal region of CfGti1 and its orthologs from *Verticillium dahliae* VdSge1 (XP_009656971.1), *Histoplasma capsulatum* Ryp1 (ABX74945.1), and *Magnaporthe oryzae* MoGti1 (XP_003713871.1). The red arrow indicates the conserved threonine residue within the potential protein kinase A phosphorylation site, and the putative nuclear localization signal is marked by asterisks. The box with the dashed line indicates the Gti1/Pac2 domains. Conserved residues are shaded in aquamarine. (**C**): Relative transcript level of *CfGti1* gene at five developmental stages including conidia, hyphae, 24 h post-inoculation (hpi), 48 hpi, and 84 hpi on apple leaves. Error bars represent the standard deviation from three technical repetitions. The data were analyzed using Tukey’s HSD test. Means associated with the same capital letter do not differ significantly (*p* > 0.05).

The protein sequences of Gti1 orthologs were downloaded from the NCBI database (https://www.ncbi.nlm.nih.gov/, accessed on 8 May 2022) (Figure 1). The BlastP tool (https://blast.ncbi.nlm.nih.gov/Blast.cgi, accessed on 13 May 2022) was used for prediction of the Gti1/Pac2 domain. All Gti1 orthologs were subjected to multiple sequence alignment by MEGA6 with default parameters. Phylogenetic analysis was conducted with MEGA version 6.06 software using the neighbor-joining method, and the statistical reliability of the tree was tested using bootstrap with 1000 replications.

### 2.2. Fungal Isolates and Culture Conditions

*Colletotrichum fructicola* wild-type strain (WT) 1104-6 was isolated from apple leaves displaying GLS symptoms in Hebei Province, China, and was stored in the laboratory of the Fungal Research Laboratory of NWAFU, Yangling, China. The isolation and pathogenicity test of 1104-6 were carried out through the following steps: Surface-sterilized pieces (5 × 5 mm) from the lesion margin of tissue were placed onto potato dextrose agar (PDA) plates. The plates were incubated at 25 °C in the dark for 5 days. Emerging fungal hyphae from the pieces were transferred to fresh PDA plates using a sterile needle to obtain pure cultures. Conidial suspensions (1 × 10^6^ conidia/mL) harvested from the pure culture were sprayed onto intact apple leaves. Inoculated leaves were placed in a humid chamber for 3–5 days. The wild-type strain and transformants generated in this study were stored as conidial suspensions at −80 °C with 30% glycerol and propagated on potato dextrose agar (PDA) plates at 25 °C.

### 2.3. Growth, Stress Tests, and Conidiation

Mycelial growth was induced on PDA at 25 °C by placing one 5 mm-diameter mycelial agar block in the center of each 90 mm Petri dish. Cell-wall stress sensitivity assays were conducted on PDA plates supplemented with either 200 μg/mL Congo red (CR) or 0.02% (*w*/*v*) sodium dodecylsulfate (SDS). The PDA plates were inoculated with mycelial plugs derived from the margin of 3-day-old colonies of WT and mutant strains. To test the impact of the pH environment on mycelial growth, the PDA plates were constructed by mixing 100 mL of 2 × PDA medium with an equal volume of adjustment buffer to achieve pH levels of 3 (0.2 M Na_2_HPO_4_ 20.4 mL plus citric acid 79.6 mL), 5 (0.2 M Na_2_HPO_4_ 51.4 mL plus citric acid 48.6 mL), or 7 (0.2 M Na_2_HPO_4_ 87 mL plus citric acid 13 mL). The diameter of mycelial colonies was recorded, and the colonies were photographed at 6 days post-inoculation (dpi). The growth inhibition ratio of mutants was calculated using the following equation.

$$\mathrm{Growth}\;\mathrm{inhibition}\;\mathrm{ratio}\left(\%\right)=\frac{\mathrm{WT}\;\mathrm{colony}\;\mathrm{diameter}-\mathrm{Mutant}\;\mathrm{colony}\;\mathrm{diameter}}{\mathrm{WT}\;\mathrm{colony}\;\mathrm{diameter}}\times 100\%$$

All assays were performed with four replicates for each strain. Conidia used for penetration and pathogenicity assays were collected as described in the literature [25].

### 2.4. Deletion of the CfGti1 Gene and Complementation Constructs

The *CfGti1* gene was deleted using the split-marker method for targeted gene replacement [36]. The upstream and downstream flanking sequences of *CfGti1* were amplified with primers LFup/LR and RF/RRdown, respectively. The hygromycin B resistance cassettes HP and PT were amplified with primers NHYGHSF/HyRNest and NygF/NHYGHSR, respectively. The HP and PT were linked with upstream and downstream sequences by overlap PCR with primers LF/XuHyR and XuYgF/RR, respectively. PEG-mediated protoplast transformation was used to introduce two resulting overlapping fragments into the WT strain 1104-6 [22]. PDA plates supplemented with 100 µg/mL hygromycin B were used to select the transformants. *CfGti1* gene deletion mutants were selected using three sets of PCR primer pairs: LF/855R, 866F/RR, and DF/DR. To analyze the homologous recombination events in the mutants, Southern blotting was conducted with the DIG-High Prime DNA Labeling and Detection Starter Kit II, Roche, catalog number 11585614910, following the manufacturer’s protocol. The probe was amplified by primers Gti1-pbF/Gti1-pbR.

For generating the *CfGti1* gene complementation construct and analyzing the cellular location of CfGti1, the open reading frame and native promoter sequence (~2 kb) of the *CfGti1* gene without a stop codon was cloned using the primers Gti1-gfpF/Gti1-gfpR. The required PCR product was linked to the vector PHZ100-GFP. The fusion expression vector PHZ100-GFP-CfGti1 was transformed into the Δ*CfGti1-15* strain. The transformants were selected on PDA medium supplemented with 300 µg/mL of geneticin. Successful complementation was confirmed by PCR with the primer pairs: LF/855R, 866F/RR, and DF/DR. Transformants were observed with a fluorescence microscope at different stages. Primers used for gene deletion, identification, and complementation are displayed in Table S1.

### 2.5. Pathogenicity Assays

Detached fully expanded leaves (cv. Gala) were drop-inoculated with 20 μL of conidial suspension (5 × 10^6^ conidia/mL) or spray-inoculated with the same concentration of conidial suspension [25]. The pathogenicity test was conducted on non-wounded apple fruit using the following method: Each apple was sprayed with a conidial suspension containing 0.1% Tween 20 (4 × 10^6^ conidia/mL). Then, all inoculated apples were incubated in a sealed plastic storage box with moist gauze on the bottom at 25 °C for 9 days. Sterile water was sprayed on the surface of the fruit every 24 h [24]. The pathogenicity test was conducted on wounded apple fruit using the following method: Each apple was wounded by a 6 mm-diameter cork borer at three sites symmetrically. Then, 20 μL conidial suspensions (5 × 10^6^ conidia/mL) of WT, deletion mutant, and revertant were inoculated at three wounded sites on one apple, respectively [25]. Inoculations were performed on three repeats (three apples) independently. To determine penetration ability, conidial suspensions were inoculated on aseptic cellophane [25].

### 2.6. RNA-Seq and qRT-PCR Validation

For RNA-seq analysis, mycelia were harvested by filtration from PDB mixed cultures as described in reference [25]. Three biological replicates were analyzed for each strain under test. To explore gene expression during leaf infection, fully expanded leaves with uniform size were sprayed until runoff with a conidial suspension (5 × 10^6^ conidia/mL). Each strain was used to inoculate four leaves, which were then incubated in moist chambers at 25 °C. Sterile water was sprayed on leaves every 24 h after inoculation in order to maintain a wet environment. At 72 h post-inoculation (hpi), when appressoria had formed, four leaves inoculated with the same strain were mixed as one sample, quick-frozen in liquid nitrogen, then sent with freezer packs to Biomarker Technologies Corporation, Beijing, China, where RNA-seq was performed using an Illumina Hi-seq X10 sequencer (Illumina, San Diego, CA, USA). The sequencing protocol was 150 bp paired-end.

The resulting clean reads were mapped to the reference genome of *C. fructicola* strain 1104-6 using TopHat (v2.1.1) software [37]. The TopHat mapping parameters were set as described in reference [25]. Transcript quantitation of each gene and identification of differentially expressed genes were calculated via the Cuffdiff 2 pipeline using default parameters. Genes with a fold change in expression > 2 and false discovery rate (FDR)-corrected *p* < 0.05 were set as down- or up-regulated. Kyoto Encyclopedia of Genes and Genomes (KEGG) and Gene Ontology (GO) analyses were performed by using the OmicShare tools (http://www.omicshare.com/tools, accessed on 25 May 2022).

Genes of interest or differentially expressed in RNA-Seq data were validated by qRT-PCR using the original samples. To monitor the expression of the *CfGti1* gene in different developmental stages, five samples (including conidia, vegetative hyphae on PDA, and apple leaves inoculated with conidia for 24 h, 48 h, and 84 h) were harvested for RNA isolation. Total RNAs were extracted using a RN53-EASYspin Plus Kit (Aidlab, Beijing, China). The resulting RNAs were used to synthesize cDNAs with a TransScript^®^ II One-Step gDNA Removal and cDNA Synthesis SuperMix (TransGen Biotech, Beijing, China, catalog number: AH311-02). qRT-PCR amplifications and relative transcript quantity were performed as described in reference [25]. Primers used for qRT-PCR assays are provided in Table S2.

## 3. Results

### 3.1. Identification and Expression of CfGti1

Two transcription factors, *CfGti1* (XP_031877756.1) and *CfPac2* (XP_031879723.1), that contain the Gti1/Pac2 domain were identified in *C. fructicola* (GenBank accession number MVNS00000000) by a local BlastP search using MoGti1 and MoPac2 in *Magnaporthe oryzae* as a query [38]. The *CfGti1* gene contains a 1359 bp open reading frame with no introns. It encoded a 452-amino-acid protein with a presumed weight of 49.5 kDa and an isoelectric point of 6.86 using the ExPASy online tool. Phylogenetic analysis revealed that CfGti1 was clustered in the same clade with other Gti1 orthologs (Figure 1A).

Sequence alignment indicated that Gti1 orthologs varied in length, and all these proteins contain a typical gluconate transport-inducing protein domain, called Gti1/Pac2 domain (Pfam09729), which is present at the N-terminus with relatively high conservation across these fungal lineages (Figure 1A). However, the C-terminus of Gti1 orthologs had relatively lower conservation. Additionally, a potential protein kinase A phosphorylation site and a putative nuclear localization signal (NLS) motif (PPGEKKR) were found in the Gti1/Pac2 domains of CfGti1 (Figure 1B).

Before testing the functions of CfGti1, we measured its transcript level. Reverse transcription–quantitative PCR (qRT-PCR) revealed that the expression of *CfGti1* was apparently higher in conidia and infection stages than that in vegetative hyphae cultivated on PDA. The transcript level of CfGti1 in early stages of infection (24 and 48 h post-inoculation, hpi) was higher than that in the late stage of infection (84 hpi) (Figure 1C). These results suggest that CfGti1 may play an important role during pathogenesis.

### 3.2. CfGti1 Is Required for Vegetative Growth, Cell Wall Integrity, and Abiotic Tolerance

**Figure 2:**
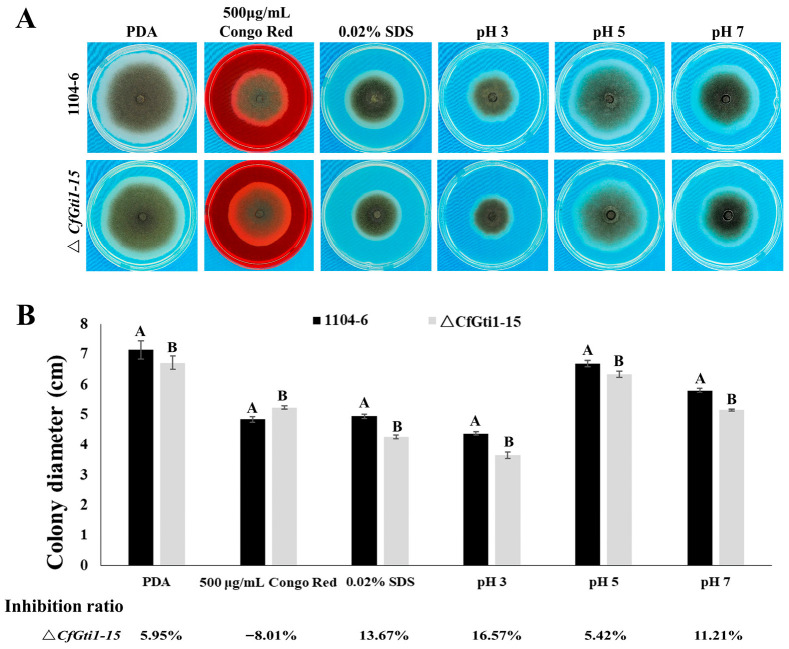
Colony morphology and stress tolerance of the Δ*CfGti1* mutant. (**A**): Colony morphology and mycelial growth on PDA medium and when supplemented with cell wall antagonists (Congo red and SDS) and different pH at 25 °C for 6 dpi. (**B**): The colony diameter indicates strains under various stresses from the colony displayed in (**A**). The inhibition ratio represents inhibition of hyphal growth compared to WT under equivalent stress conditions. Error bars represent the standard deviation from four independent repetitions; clustered means associated with the different capital letters are significantly different (*p* ≤ 0.05) using Tukey’s HSD test. PDA, potato dextrose agar; SDS, sodium dodecylsulfate.

To assess the role of *CfGti1* in *C. fructicola*, the *CfGti1* gene knock-out mutant strain Δ*CfGti1-15* was generated using a split-marker approach (Figure S1A). A deletion mutant was identified by three PCR reactions and confirmed by Southern blot analysis (Figure S1B,C). For complementation of Δ*CfGti1-15*, the *CfGti1* gene with its native promoter region (~2 kb) was transformed into Δ*CfGti1-15*. The complementary strain CfGti1-15C10 was confirmed by three PCR reactions (Figure S1B). The colony of both wild type (WT) and Δ*CfGti1-15* displayed black in an inner ring and white in an outer ring (Figure 2A). For the vegetative growth test, the colony diameter of Δ*CfGti1-15* showed a 6% reduction compared to WT 1104-6 on PDA plates after 6 days of incubation.

To evaluate sensitivity to cell wall antagonists (SDS and Congo red), the WT and Δ*CfGti1-15* were cultivated on PDA supplemented with 0.02% SDS or 500 μg/mL Congo red, respectively. The Δ*CfGti1-15* showed a 13.7% reduction in colony diameter on PDA with 0.02% SDS and an 8% increase on PDA supplemented with Congo red. On PDA of pH 5, Δ*CfGti1-15* showed growth reduction just like the vegetative growth test, compared to 16.6% and 11.2% reduction at pH 3 and 7, respectively (Figure 2A,B). These results indicate that CfGti1 plays a role in maintaining cell wall integrity and pH tolerance.

### 3.3. CfGti1 Is Indispensable for Pathogenicity and Colonization of C. fructicola

**Figure 3:**
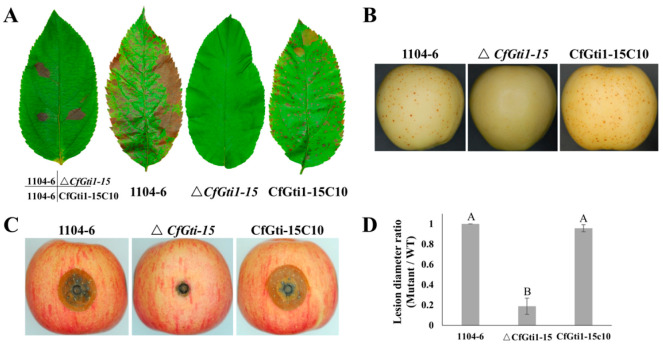
Pathogenicity defects of Δ*CfGti1* mutant on apple leaves and fruit (cv. Gala). (**A**): Pathogenicity defects of Δ*CfGti1* mutant on apple leaves at 25 °C for 5 d. (**B**): Pathogenicity test of WT and mutant strains on non-wounded apple fruit at 25 °C for 9 dpi. (**C**): Pathogenicity test of WT and mutant strains on wounded apple fruit at 25 °C for 7 dpi. (**D**): Lesion diameter ratio of Δ*CfGti1* mutant compared to WT on wounded fruit at 7 dpi. Error bars represent the standard deviation from three independent repetitions. Means associated with the same capital letters to not differ significantly (*p* > 0.05) using the LSD test.

To determine if CfGti1 is involved in pathogenicity, we performed pathogenicity tests on detached apple leaves and fruit (cv. Gala). By drop inoculation of conidial suspensions side by side on leaves, the WT 1104-6 and the complementary strain CfGti1-15C10 caused conspicuous black necrotic spots at 5 days post-inoculation (dpi). Under the same conditions, however, the Δ*CfGti1-15* mutant did not induce any lesions (Figure 3A). Spray-inoculation of the WT and CfGti1-15C10 conidial suspensions on leaves caused massive lesions on leaves, compared to no lesions for spray-inoculation with Δ*CfGti1-15* (Figure 3A). Spraying apple fruit with a conidial suspension of Δ*CfGti1-15* caused no necrotic spots, whereas spraying WT and CfGti1-15C10 inoculum caused numerous lesions of about 1–2 mm in diameter (Figure 3B). These results indicate that *CfGti1* is indispensable for pathogenicity of *C. fructicola*.

To investigate the function of CfGti1 in colonization and hyphal extension, appressorium-mediated penetration was bypassed by wound inoculation. A 20 μL conidial suspension of each strain was inoculated into wounded apple fruit. At 7 dpi, lesion diameter caused by Δ*CfGti1-15* on wounded fruit averaged 92 ± 2% smaller than for inoculation with the WT. At the same time, lesion diameter caused by CfGti1-15C10 was 96 ± 14% of that caused by the WT (Figure 3C,D). This result indicates that Δ*CfGti1* was almost entirely deficient in colonization of wounded apple fruit tissue.

### 3.4. CfGti1 Is Involved in Penetration and Hyphal Development After Penetration

**Figure 4:**
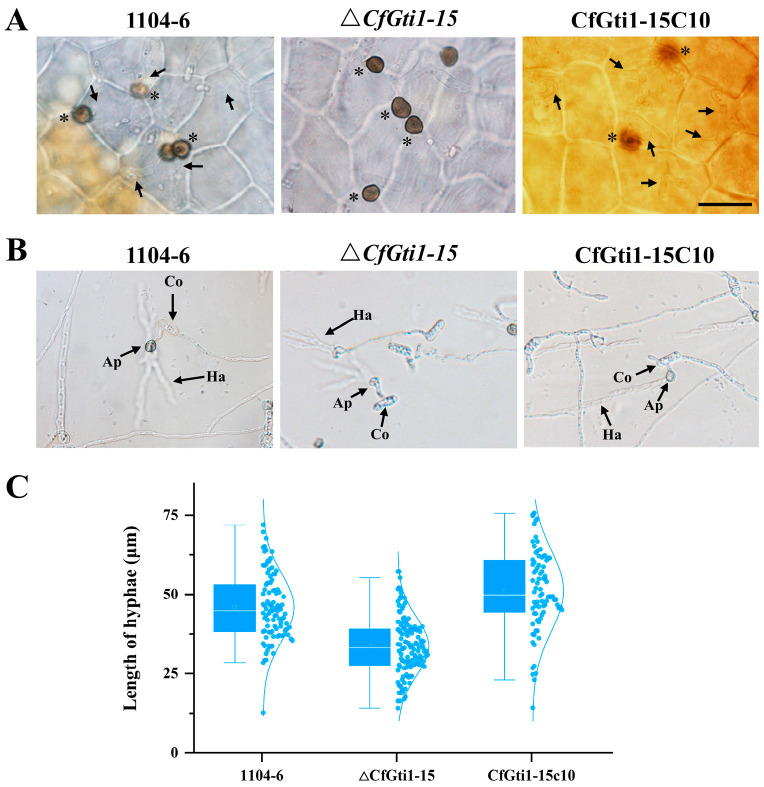
Penetration and hyphal development defects of the Δ*CfGti1* mutant. (**A**): Penetration defect of the Δ*CfGti1* mutant on apple leaves at 4 dpi; leaves have been decolorized to aid in viewing the pathogen. Asterisks indicate appressoria formed by strains on the leaf surface. Black arrows indicate invasive hyphae under appressoria in leaf cells. Bar = 20 μm. (**B**): Hyphal development defects of the Δ*CfGti1* mutant after penetrating cellophane at 25 °C for 18 h after conidia inoculation. Co: conidium; Ap: appressorium; Ha: hyphae after penetration. Bar = 20 μm. (**C**): Box chart shows the length of hyphae under cellophane after penetration at 25 °C for 18 hpi displayed in (**B**).

To determine the stage at which the *CfGti1* gene deletion mutant was halted during leaf infection, inoculated leaves were examined microscopically. At 4 dpi, all tested strains including Δ*CfGti1-15* formed massive numbers of appressoria on the leaf surface. Although the WT and complementary strains developed hyphae in leaf cells, Δ*CfGti1-15* failed to penetrate leaf epidermal cells (Figure 4A). However, Δ*CfGti1-15* readily penetrated cellophane, although developing abnormally shortened hyphae after penetration. The complemented strain CfGti1-15C10 penetrated leaf tissue in the same manner as the WT (Figure 4B,C).

### 3.5. Comparative Transcriptome Analysis Revealed Genes Modulated by CfGti1

**Figure 5:**
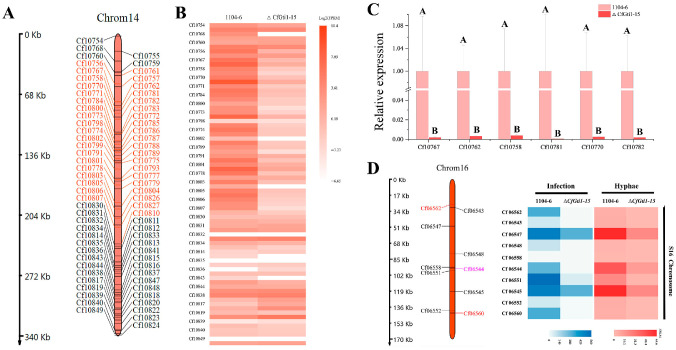
The expression pattern of genes on accessory chromosomes was affected after deletion of *CfGti1*. (**A**): Gene distribution map of the 14th accessory chromosome according to third-generation genome sequencing and assembly. Genes shown in red were sharply down-regulated in Δ*CfGti1-15* compared to WT in vegetative hyphae. (**B**): The heat map indicates the gene transcript levels of the 14th accessory chromosome based on the FPKM value of vegetative hyphae from three technical repetitions. (**C**): Fluorescence qPCR of six selected genes was used to verify the transcriptome data of the 14th accessory chromosome. Error bars represent the standard deviation from three technical repetitions. Clustered means associated with different capital letters differ significantly (*p* ≤ 0.05). (**D**): Gene distribution of the 16th accessory chromosome and gene transcript levels of infection (72 hpi on apple leaves) and vegetative hyphae. Two genes with red color are salicylate synthetase ybtS and MaSalS ortholog; the gene with purple color is acyl-CoA ligase AKT1 and AFT1-1 ortholog. The transcriptome data of infection were derived from a pooled sample of four independent samples, while the transcriptome data of vegetative hyphae were obtained from three independent biological replicates.

To uncover the underlying mechanisms of the phenotype and pathogenicity defection in the Δ*CfGti1* mutant, we performed transcriptome sequencing (RNA-seq) analysis. Compared to the WT, 1248 genes showed differential expression (fold change ≥ 2, Q-value < 0.05) in mycelia of the Δ*CfGti1-15* mutant. Among those genes, 570 and 678 genes were up- and down-regulated, respectively. Results of reverse transcription–quantitative PCR (qRT-PCR) of six down-regulated genes were consistent with RNA-seq data (Figure 5C). During leaf infection, 2008 and 1937 genes were up- and down-regulated (fold change ≥ 4), respectively, in the Δ*CfGti1-15* mutant compared to the WT.

Gene Ontology (GO) enrichment analysis showed that most affected genes associated with mycelial growth were related to metabolism, catalytic activity, and single-organism processes (Figure S2A). Down-regulated genes were enriched in the membrane, the intrinsic component of the membrane, and the integral component of the membrane (Figure S2B). A KEGG pathway enrichment analysis revealed that down-regulated genes were enriched in ether lipid metabolism, nicotinate and nicotinamide metabolism, or butanoate metabolism pathways (Figure S2C).

**Figure 6:**
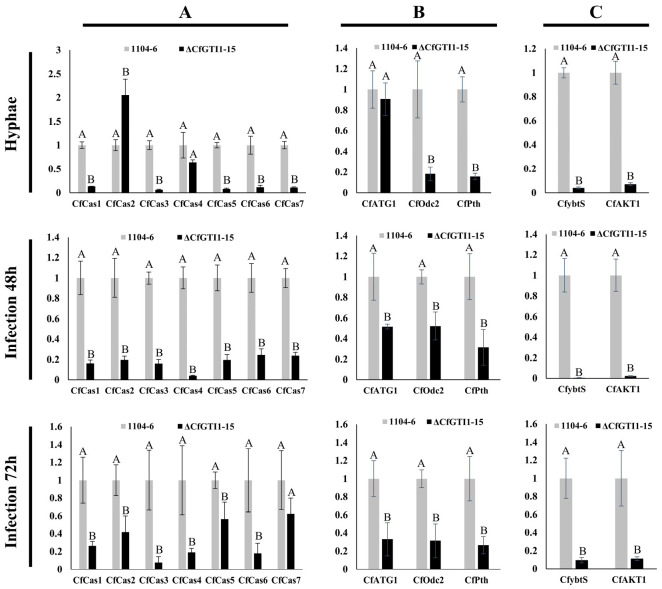
Fluorescence qPCR of orthologues of pathogenicity-related genes during vegetative hyphae, leaf infection at 48 h, and leaf infection at 72 h in *C. fructicola*. (**A**): Relative expression of seven Gas1-like DUF3129 family genes that are required for appressorium-mediated penetration in three phases. (**B**): Relative expression of three penetration-related genes in three phases. (**C**): Relative expression of two putative virulence-related genes that reside in 16th accessory chromosomes in three phases. Error bars represent the standard deviation from three technical repetitions. Clustered means associated with the same capital letters do not differ significantly (*p* > 0.05).

As mentioned above, deletion of the *CfGti1* gene led to defect of appressorium-mediated penetration on leaves. Consistent with this defect, many genes involved in appressorium-mediated penetration were strongly down-regulated in Δ*CfGti1-15* during leaf infection, including the serine/threonine kinase gene *CfATG1* (*MgATG1* ortholog) [39], peroxisomal carnitine acetyl transferase gene *CfPTH2* (*PTH2*) [40], oxalate decarboxylase gene *CfOdc2* (*Ss-odc2*) [41], and four of seven Gas1-like DUF3129 family genes, *CfCas3*, *CfCas4*, *CfCas5*, and *CfCas6* [42] (Table 1). The relative expression of Gas1-like DUF3129 family genes (*CfCas1*–*CfCas7*), *CfATG1*, *CfPTH2*, and *CfOdc2* genes was verified by qRT-PCR in three phases. All genes mentioned above were down-regulated in Δ*CfGti1-15* during production of hyphae, leaf infection (48 h), and leaf infection (72 h) except *CfCas2*, *CfCas4*, and *CfATG1* in hyphae, and *CfCas7* in infection (72 h) (Figure 6A,B).

Expression of multiple pathogenesis-related genes was affected by the deletion. Among them, a multidrug resistance transporter *Cf07568* (*CgTpo1_2*) [43], a major facilitator superfamily transporter *Cf08985* (*CaNAG4*) [44], a tensin-like phosphatase *Cf15556* (*FgTep1*) [45], and eisosome *Cf09682* (*PilB*) [46] were down-regulated during both leaf infection and vegetative hyphae in Δ*CfGti1-15* (Table 1). In addition, kinase *Cf00781* (*FgCtk1*) [47], pyruvate dehydrogenase kinase *Cf16115* (*FgPDK1*) [48], bZIP transcription factor *Cf07907* (*GzbZIP007*) [49], ferric reductase *Cf10343* (*FreB*) [50], secreted LysM Protein 1 *Cf05289* (*Slp1*) [51], effector candidates *Cf02031* (*ChEC91*) [52], retromer component *Cf07959* (*FgVps29*) [53], pectate lyase *Cf16857* (*pelB*) [54], methylenetetrahydrofolate reductase *Cf16072* (*MET13*) [55], α-1,2-mannosyltransferase *Cf00854* (*Ktr4*) [56], ubiquitin-conjugating enzyme *Cf09590* (*FgPEX4*) [57], exosome component *Cf00756* (*GzOB047*) [49], ROGDI domain contain gene *Cf11008* (*FgRav2*) [58], and Ca^2+^-ATPases *Cf00193* (*MGG_05078.5*) [59] were down-regulated during leaf infection in Δ*CfGti1-15* (Table 1).

**Table 1:** Expression of differentially expressed genes in comparative transcriptome analysis.

Gene ID	Gene Name	Accession Number	Description	Leaf Infection			Hyphae			References
				FPKM (1104-6)	FPKM (Δ*CfGti1-15*)	Log_2_ (FC)	FPKM (1104-6)	FPKM (Δ*CfGti1-15*)	Log_2_ (FC)	
Cf15211	CfCas1	XP_031876572.1	DUF3129 domain protein	565.9	389.8	−0.5	3.7	8.5	1.2	[42,60]
Cf17702	CfCas2	XP_031887373.1		69.0	247.9	1.8	4.5	5.8	0.4	
Cf02102	CfCas3	XP_031881140.1		1186.6	634.8	−0.9	0.0	0.2	0.0	
Cf08259	CfCas4	XP_031885643.1		174.1	21.8	−3.0	38.6	38.5	0.0	
Cf04403	CfCas5	XP_031892225.1		378.8	0.0	—	2.7	4.2	0.6	
Cf09988	CfCas6	XP_031882861.1		44.0	0.0	—	3.1	4.9	0.6	
Cf16160	CfCas7	XP_031893297.1		116.3	206.8	0.8	53.1	18.1	−1.6	
Cf02383	CfAtg1	XP_031886481.1	Serine/Threonine protein kinase	43.0	7.4	−2.5	77.9	118.0	0.6	[39]
Cf07707	CfPth2	XP_031885495.1	Peroxisomal carnitine acetyl transferase	86.2	0.0	—	36.7	33.3	−0.1	[40]
Cf05405	CfOdc2	XP_031881493.1	Oxalate decarboxylase	151.4	0.0	—	27.8	1.2	−4.6	[41]
Cf07568	CfTpo1	XP_031879473.1	Major facilitator superfamily transporter	64.6	28.5	−1.2	142.7	7.0	−4.3	[43]
Cf08985	CfNag4	XP_031877042.1	Major facilitator superfamily transporter	217.9	87.4	−1.3	187.6	12.7	−3.9	[44]
Cf15556	CfTep1	XP_031877149.1	Tensin-like phosphatase	565.2	268.7	−1.1	224.2	28.7	−3.0	[45]
Cf09682	CfPilB	XP_031882758.1	Sphingolipid long chain base-responsive	712.1	494.9	−0.5	212.9	47.6	−2.2	[46]
Cf00781	CfCtk1	XP_031880716.1	Ctd kinase	115.4	10.3	−3.5	32.4	45.9	0.5	[47]
Cf16115	CfPDK1	XP_031876717.1	Pyruvate dehydrogenase kinase	294.0	0.0	—	89.6	76.1	−0.2	[48]
Cf07907	CfZip007	XP_031882559.1	bZIP transcription factor	215.7	0.0	—	140.3	164.7	0.2	[49]
Cf10343	CfFreB	XP_031884339.1	Ferric reductase	133.4	14.7	−3.2	55.5	53.5	−0.1	[50]
Cf02031	CfEC91	XP_031881071.1	Hypersensitive response-inducing protein	341.2	0.0	—	15.9	3.5	−2.2	[52]
Cf07959	CfVps29	XP_031883282.1	Vacuolar protein sorting-associated protein	212.1	0.0	—	44.0	53.4	0.3	[53]
Cf16857	CfPelB	XP_031889329.1	Pectate lyase	148.7	0.0	—	67.3	268.6	2.0	[54]
Cf16072	CfMet13	XP_031885300.1	Methylenetetrahydrofolate reductase	117.1	0.0	—	52.1	71.4	0.5	[55]
Cf00854	CfKtr4	XP_031887124.1	α-1,2-mannosyltransferase	116.9	0.0	—	84.0	86.0	0.0	[56]
Cf09590	CfPEX4	XP_031888472.1	Ubiquitin-conjugating enzyme	106.7	0.0	—	76.0	98.4	0.4	[57]
Cf00756	CfGzOB047	XP_031891276.1	Exosome component exosc1 csl4	94.4	0.0	—	18.5	15.8	−0.2	[49]
Cf11008	CfRav2	XP_031881664.1	ROGDI domain contain protein	90.4	0.0	—	19.4	18.7	−0.1	[58]
Cf00193	CfATPase3	XP_031888621.1	Calcium-transporting ATPase 3	121.0	19.4	−2.6	28.2	22.8	−0.3	[59]

### 3.6. CfGti1 Modulates the Gene Expression Residing on Accessory Chromosomes of C. fructicola

In contrast to the core chromosomes, accessory chromosomes (generally less than 2.0 MB) which integrate numerous transposable elements and pathogenicity genes are dispensable for normal growth but essential for determining host-specific virulence [61,62].

After acquisition of a high-quality genome by long-read sequencing and Hi-C map data, we found that there are five putative accessory chromosomes (0.17 MB to 0.92 MB) in *C. fructicola* [63]. In the 14th putative accessory chromosome (0.35 MB), a predicted 82 genes resided there (Figure 5A). Based on transcriptome data of vegetative hyphae, 45 adjacent genes were drastically down-regulated because of deletion of *CfGti1* (Figure 5A,B). Among them, six genes were selected to verify the transcript levels by qRT-PCR; this result was consistent with transcriptome data (Figure 5C). In addition, 10 predicted genes residing on the 16th putative accessory chromosome (0.17 MB) were also all down-regulated, not only in vegetative hyphae growth but also in leaf infection in Δ*CfGti1-15* (Figure 5D). Nine of these genes were induced during leaf infection. Of them, two presumed virulence-related genes were identified: a salicylate synthetase gene *CfytbS* (*Cf06562*, *Cf06560*) and an acyl-CoA ligase gene *CfAKT1* (*Cf06544*). The *CfytbS* was a *ybtS* and *MaSalS* ortholog, which were required for virulence in *Klebsiella pneumonia* and *Metarhizium acridum*, respectively [64,65]. The *CfAKT1* was an *AKT1* and *AFT1-1* ortholog, which were required for biosynthesis of AK and AF host-specific toxin, respectively, in *Alternaria alternata* [66,67]. The relative expression of *CfytbS* and *CfAKT1* was verified by qRT-PCR and dramatically down-regulated for vegetative hyphae growth, leaf infection at 48 h, and leaf infection at 72 h (Figure 6C).

### 3.7. CfGti1-GFP Localizes to the Nucleus

**Figure 7:**
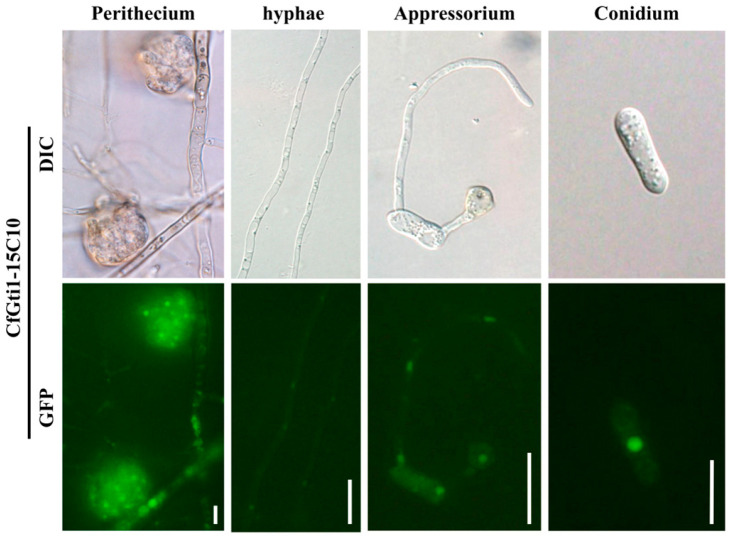
Subcellular localization of CfGti1-GFP. Fluorescence microscopy of CfGti1-GFP in conidia, appressoria, hyphae, and perithecia. Conidia and hyphae were harvested from 4-day-old PDB cultures. Bar = 20 μm. Appressoria were observed on cellophane 18 h after inoculation with a conidial suspension. Bar = 20 μm. Perithecia were obtained from 7-day-old oatmeal agar cultures. Bar = 20 μm. The fluorescence of protein was localized mainly in the nucleus.

To analyze the cellular location of CfGti1, a CfGti1-GFP construct was generated and transformed into the mutant Δ*CfGti1-15*. The resulting transformant CfGti1-15C10 was screened by geneticin, PCR reactions, and GFP fluorescence. Under examination by fluorescence microscopy, weak GFP fluorescence signals were detected exclusively in the nuclei of conidia, appressoria, hyphae, and perithecia. In hyphae, the GFP fluorescence signal intensity seemed weaker than for other cell types (Figure 7). This result was consistent with gene expression analysis of *CfGti1*. These data indicate that CfGti1-GFP possessed characteristics of a transcription factor and was localized to the nucleus in *C. fructicola*.

## 4. Discussion

In the present study, we characterized a key pathogenicity-related transcription factor in *C. fructicola*, *CfGti1*, which possesses pleiotropic functions in pathogenicity and colonization by regulating infection-related morphogenesis, including host penetration and hyphae development. These results provide important new insight into the genetic regulation of GLS pathogenesis.

Gti1 and its ortholog’s transcription factor were vital for morphological switching and pathogenesis in the human fungal pathogen *Candida albicans* and *Histoplasma capsulatum*. Specifically, it was required for switching two different cell types when encountering specific environmental transformation [68,69,70]. In many plant-pathogenic fungi, Gti1-like proteins are also involved in virulence. In *Fusarium verticillioides*, *F. graminearum*, *Zymoseptoria tritici*, and *Cladosporium fulvum*, Gti1-like gene deletion mutants induced disease symptoms with reduced virulence [71,72,73,74]. In *F. oxysporum*, *Botrytis cinerea*, and *M. oryzae*, Gti1-like gene deletion affected host penetration either weakly or not at all [75,76,77]. In contrast, *CfGti1* gene deletion in *C. fructicola* resulted in total loss of GLS symptoms due to its shutdown of host penetration. These observations showed that CfGti1 performs essential roles in pathogenicity of *C. fructicola*.

The deletion mutant Δ*CfGti1* resulted in loss of *C. fructicola*’s ability to penetrate a plant host, but it was still able to penetrate cellophane. These results suggest that Δ*CfGti1* retained the ability, at least in part, to develop a penetration peg. Two completely different penetration phenotypes on two different surfaces may reflect the defects of Δ*CfGti1* in host plant signal recognition or mechanical penetration pressure. Consistent with this phenotype of host penetration defect, we identified several appressorium-mediated, penetration-related genes that were putatively regulated by CfGti1, including a serine/threonine kinase MgATG1 orthologous gene [39], a peroxisomal carnitine acetyl transferase PTH2 orthologous gene [40], an oxalate decarboxylase Ss-odc2 orthologous gene [41], and a Gas1-like DUF3129 family of genes [42]. Of these genes, MgATG1 is indispensable for initiating autophagy and lipid turnover in conidia and appressoria, which is essential for appressorium-mediated host penetration [39], and Gas1-like DUF3129 proteins are enriched in appressoria and promote appressorial penetration by promoting lipid droplet degradation [42]. The impairment of appressorium-mediated penetration of the *PTH2* gene deletion mutant was due to delayed mobilization of lipid droplets in infection structures [40]. Future research should aim to determine whether CfGti1 regulates appressorium-mediated host penetration by targeting various penetration-related genes, especially those related to lipid droplet metabolism.

When the penetration process was bypassed by wound inoculation of apple fruit, the Δ*CfGti1* mutant was almost unable to colonize fruit tissues. Similar colonization loss was also observed in the deletion mutant of *VdSge1* (*Gti1* ortholog) in *V. dahliae* [78]. In *Cladosporium fulvum,* deletion of a Gti1-like gene affected only part of the colonization process [74]. In contrast, the Gti1-like protein is not required for host colonization in *F. verticillioides* and *F. oxysporum* [71,75]. Overall, the functions that Gti1-like proteins perform in colonization vary among plant pathogenic fungi. Our results suggest that CfGti1 is indispensable for colonization by *C. fructicola.* A hypothesized explanation for loss of colonization ability by Δ*CfGti1* is deficient development of hyphal growth and development. At the level of gene regulation, we identified numerous virulence-related genes that were deactivated during vegetative hyphae growth and infection in Δ*CfGti1*. These results suggest that CfGti1 performs functions in colonization and virulence by modulating virulence-related genes.

Among pathogenic fungal species, accessory chromosomes that are generally small and harbor pathogenicity-related genes exist separately from the core chromosomes and play a prominent role in pathogenicity in some cases [79,80]. Accessory chromosomes are notable in their ability to transform a non-pathogenic strain into a pathogenic strain by means of horizontal transfer of these chromosomes between strains [81,82]. In the present study, CfGti1 acted as a regulator for genes residing on accessory chromosomes of *C. fructicola*. Interestingly, two putative pathogenicity-related genes, a salicylate synthetase gene (*ybtS* orthologue) and an acyl-CoA ligase gene (*AKT1* and *AFT1-1* orthologue), residing on an accessory chromosome of *C. fructicola,* are also modulated by CfGti1. AKT1 and AFT1-1 are responsible for biosynthesis of host-specific toxins AK and AF, which share many aspects of their molecular structure [66,67]. The *ybtS* gene encodes salicylate synthetase, which is required for the early steps of the biosynthesis of yersiniabactin, which is involved in iron uptake and pathogenesis of pneumonic plague and bubonic plague [83]. Studies about the pathogenicity-related accessory chromosomes were relatively indistinct in *Colletotrichum* species. In the future, it is worth exploring whether there is linkage between virulence-related genes residing on accessory chromosomes, toxin synthesis, and pathogenesis of GLS.

## 5. Conclusions

Taken together, our results demonstrate that CfGti1, a key pathogenic-related transcription factor, is essential for regulating appressorium-mediated host penetration, colonization, hyphal extension, and pathogenesis in *C. fructicola*.

Methodological Limitation: These transcriptomic analyses were performed using the then-standard TopHat/Cuffdiff pipeline. We note that the rapid evolution of RNA-seq analysis means newer tools (e.g., HISAT2/StringTie for alignment/assembly or Salmon/DESeq2 for quantification) now offer improvements in accuracy and computational efficiency.

## Data Availability

The original contributions presented in this study are included in the article. Further inquiries can be directed to the corresponding authors.

## Supplementary Materials

The following supporting information can be downloaded at: https://www.mdpi.com/article/10.3390/jof12010036/s1, Figure S1: Targeted gene replacement and *CfGti1* gene deletion mutant identification; Figure S2: Function categorization of the differentially expressed genes in the Δ*CfGti1* mutant. Table S1: Primers used for gene knockout, mutant identification and complementation. Table S2: qRT-PCR primers used in this study.
