# Supplementary material for: Colletotrichum fructicola CfGti1 Transcriptionally Regulates Penetration, Colonization, and Pathogenicity on Apple
Source: J Fungi (Basel). 2026 Jan 2;12(1):36. doi: 10.3390/jof12010036 (PMC12842837; doi:10.3390/jof12010036)
Supplement: Supplementary file 1 [file jof-12-00036-s001.zip › jof-4060574-supplementary.pdf]

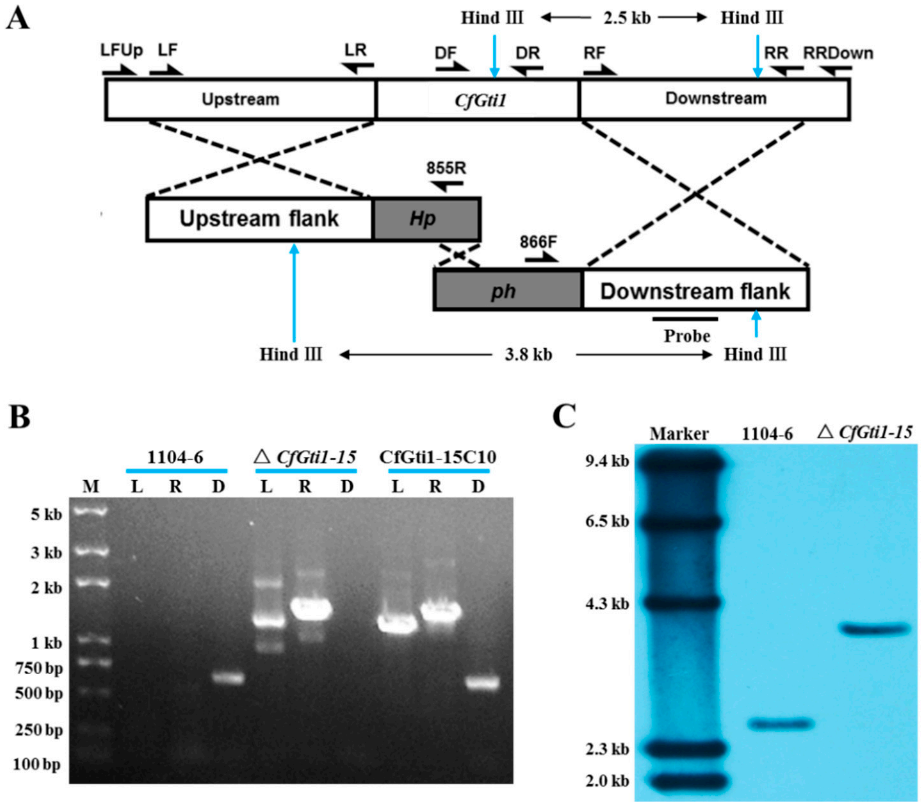


**Figure S1.** Targeted gene replacement and *CfGti1* gene deletion mutant identification. A: Schematic representation of *CfGti1* gene deletion strategy. Black arrows represent primers, black boxes (Hp, ph) are two split fragments of hygromycin phosphotransferase gene. Light blue arrows represent Hind III restriction enzyme cutting site. Black bar represents probe used for southern blot. B: PCR amplification analysis was used to identify gene deletion strain with three sets of primer pairs. M: DNA marker L: LF/855R (1266 bp) R: 866F/RR (1379 bp) D: DF/DR (617 bp). C: Southern hybridization analysis was used to validate the deletion of *CfGti1* genes with probe indicated in figure S1A.

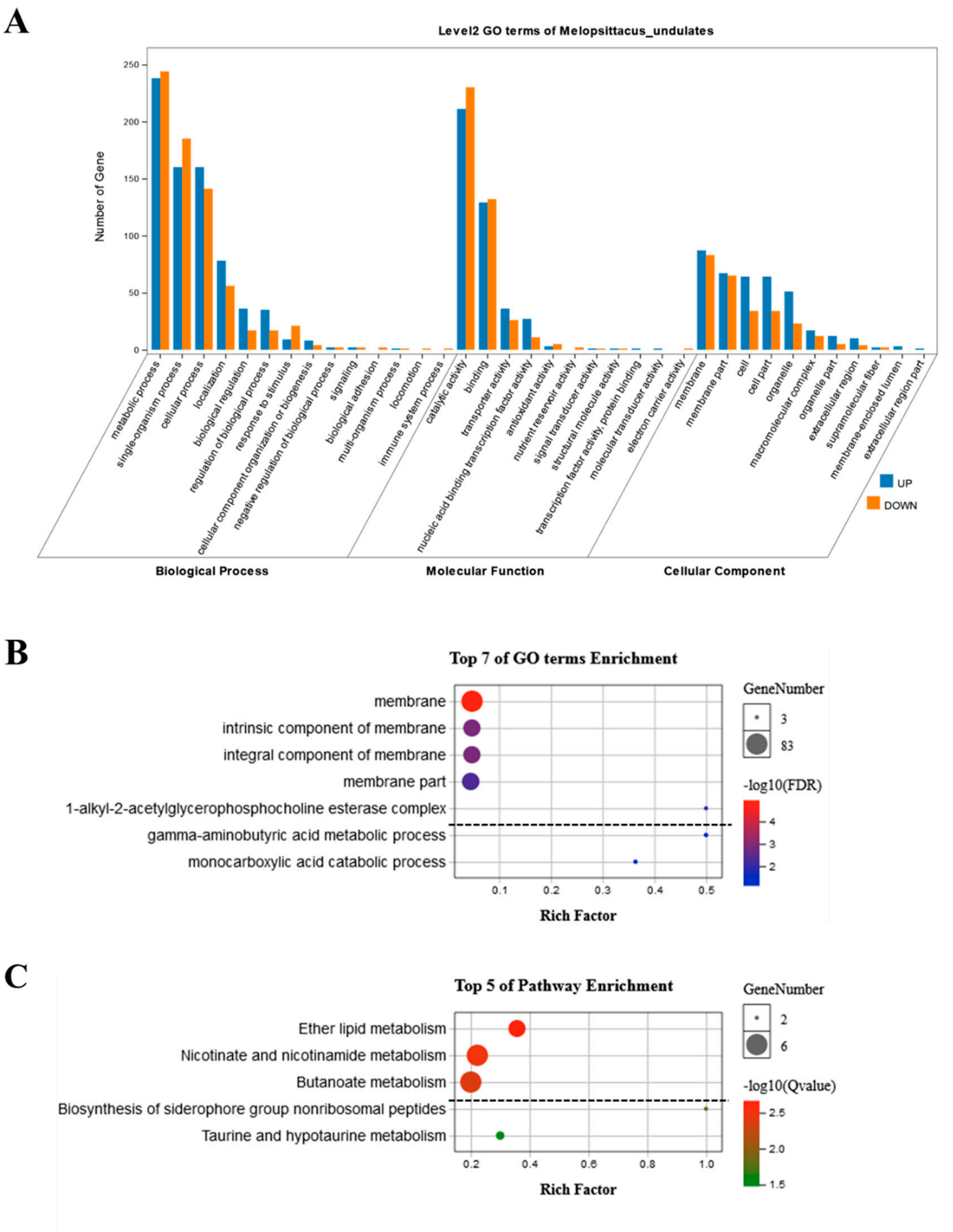


**Figure S2.** Function categorization of the differentially expressed genes in the Δ*CfGti1* mutant. A: Functional grouping of genes up- or down-regulated in the Δ*CfGti1* mutant. Genes with more than 2-fold change were grouped based on Gene Ontology (GO) annotations and their putative functions. B: Bubble diagram indicates GO enrichment analysis of the Δ*CfGti1* mutant 2-fold-downregulated genes compared to WT. Rich Factor represents the ratio of down-regulated genes in this pathway among all genes in this pathway. GO terms above the dotted line are Q value < 0.01. C: KEGG pathway enrichment analysis of 2-fold-downregulated genes in the Δ*CfGti1* mutant. The pathway rich factor represents the ratio of downregulated genes in this pathway among all genes in this pathway. The top 5 enriched pathways are listed. KEGG terms above the dotted line are Q value < 0.01.

**Table S1** Primers used for gene knockout, mutant identification and complementation

| Primer name | Primer sequence |
|---|---|
| Gti1-LFup | GAGCCCGATCTGCCCTGTACTTGAC |
| Gti1-LF | TGCTGCTCCTCTGGTTCTGCTACT |
| Gti1-LR | AGACTGTGTGGGATCTAGTCTGACC |
| Gti1-RF | CACCTGCCACTGACAGTAGTCCGAT |
| Gti1-RR | CGTTGTTAGTCAGGTAGTTCGCTCC |
| Gti1-RRdown | TACCTTGTACTCTTCCCATCGCTTC |
| Gti1-DF | CGACCGTGAAAGACAGGACCTCATC |
| Gti1-DR | GGTGACTATTGACGGGATACGGATG |
| Gti1-gfpF | ACCGCGGTGGCGGCCGCGACACACTACGGATACAGACTCACG |
| Gti1-gfpR | GCTCACCCTATCGAATTCAGTCCTATTCATGGCGTTGCTCCCG |
| HyRNest | AGAGTTGGTCAAGACCAATGC |
| NHYGHSF | AGTCGACGACAACTACCATCGATCTGACGGTCGACAGAAGATGATATTGAAGGA |
| NygF | CGAAAAGTTCGACAGCGTCTC |
| NHYGHSR | ACACTGGTGACGGCTAACCAGAACTGTCAGAAGAGGTAAACCCGAAACGC |
| XuHYR | GTATTGACCGATTCCTTGCGGTCCGAA |
| XuYGF | GATGTAGGAGGGCGTGGATATGTCCT |
| Xu855R | GCTGATCTGACCAGTTGCCT |
| Xu866F | GTCGATGCGACGCAATCGT |
| HY | CGCCCTTCCTCCCTTTATTTC |
| YG | TGTCGTCCATCACAGTTTGCC |
| Gti1-pbF | TCTGAAGAGAACGGCTGGATGGAG |
| Gti1-pbR | GCTGAATTGCCTTGGTCGGAAGAG |

**Table S2** qRT-PCR primers used in this study

| Gene ID | Accession number | Forward Primer | Reverse Primer |
|---|---|---|---|
| Cf10782 | XP_031892222.1 | CGAACAGCGGAATCAGACAATCACA | GTCCACACTCAGAGCACCAGTAACA |
| Cf10781 | XP_031875974.1 | GGAAGACGGCTGCGAAGATGCT | AAGTGGCGACTGTTGAAGAGGAGAC |
| Cf10762 | XP_031877233.1 | CTGGCGACAGTAGGCTGCTGATTG | CACATCTCCTCCGTCCTCTCCTTCC |
| Cf10758 | XP_031881202.1 | GGAATCAGACAGACACTGCGGAGAT | CCGTCAGGCTCACATCCATTACAG |
| Cf10770 | XP_031880558.1 | ATCGTCGTCTTCGCAACAGGCTAC | CATTAGGCAGTCCGTCAGCAACCAT |
| Cf10767 | — | GCCTTGAACAACAACAGCAACATACG | CCACCAGAGATACAGGTAGCCAAGC |
| CfGti1 | XP_031877756.1 | CCGTCAATAGTCACCACCAGAGCAT | CCCAGGCACCGCACTCAAATACT |
| CfPac2 | XP_031879723.1 | AGTCGTGGAGTGCCAGCAGAGT | CTTCATCAGACCGTCCGCCTTGTAT |
| CfCas1 | XP_031876572.1 | TCGGTGGTGGTGGCAAGAACAAG | TGGCGGAAGGTCATGGTGATGGTA |
| CfCas2 | XP_031887373.1 | TCGACAGTGTTCAGGGTGCCAAC | TCACGAGGAGTGCCGTCGATCAA |
| CfCas3 | XP_031881140.1 | GTGCCACCTGGACCAACATCAAC | GCAGCGGACCAAGCAGACATTG |
| CfCas4 | XP_031885643.1 | GCCGTCCAGCAATCCGATGTCA | CTTGTTGGCAGCAGCGATGTTGTC |
| CfCas5 | XP_031892225.1 | AAGGTCCAGGTTGCCGACATCG | GGTGGTGACGACGTGGAAGGTT |
| CfCas6 | XP_031882861.1 | GTGGCTTCGGCTCAGGGTGTTATC | CGTTGTCGGTGATCTCCTTGGTGTT |
| CfCas7 | XP_031893297.1 | TGCTGGTGGTCAGGGTATGGCTATC | TATTGGTGCCTCCGCCGACAGT |
| CfAtg1 | XP_031886481.1 | GTCTTCGCTGTCTCGCCGCTTATC | GCCTTCACTCGGACCTGACAACCT |
| CfPth2 | XP_031885495.1 | CTTGTGCTTGTTGTCAGGACGGATCT | AGAGAATCTTGGCGGCAGCAGAAC |
| CfOdc2 | XP_031881493.1 | ATCCAAGAGCAGAACCAGACCTCCT | GACAACAACCAGAGCGGCAGAGAA |
| CfybtS | XP_031879412.1 | GTGACTGCGACTGGTGTTCCGAA | CACTGGCGAGACTGCTGCTGAA |
| CfAKT1 | XP_031877688.1 | TGCGGCGGCTACTGTCGGAATA | GTCGTTTGCCTTGTCCTGATGTCCAA |
